# Neuropsychological Tests for Detecting Prodromal DLB: A Systematic Review

**DOI:** 10.1111/ene.70681

**Published:** 2026-07-10

**Authors:** Francesco Mori, Fabrizia D'Antonio, Giulia Zazzaro, Desirée Conti, Micaela Mitolo, Annachiara Cagnin, Annalena Venneri

**Affiliations:** ^1^ Department of Medicine and Surgery University of Parma Parma Italy; ^2^ Department of Clinical and Experimental Sciences University of Brescia Brescia Italy; ^3^ Department of Human Neurosciences Sapienza University of Rome Rome Italy; ^4^ Cognitive and Motor Rehabilitation and Neuroimaging Unit IRCCS Fondazione Santa Lucia Rome Italy; ^5^ Department of Psychology Sapienza University of Rome Rome Italy; ^6^ IRRCS Istituto delle Scienze Neurologiche Bologna Italy; ^7^ Department of Neuroscience and Padua Neuroscience Center University of Padua Padua Italy; ^8^ Department of Neuroscience University of Sheffield Sheffield UK

**Keywords:** MCI, neuropsychological profile, neuropsychological tests, prodromal DLB

## Abstract

**Background:**

The prodromal phase of Dementia with Lewy bodies (DLB) remains unexplored and poorly defined. This systematic review scrutinised studies on the earliest cognitive DLB deficits, focusing on the most useful neuropsychological tests.

**Methods:**

From an initial pool of 2307 articles, 37 articles met inclusion criteria including either a prodromal DLB population or DLB cohorts comprehensive of individuals in prodromal or Mild Cognitive Impairment (MCI) due to Lewy Bodies pathology. These studies included comparisons with healthy controls, MCI due to Alzheimer's Disease (MCI‐AD), or other aetiologies, and patients with established dementia.

**Results:**

Mild impairments were found in attention, executive functions, visual perception, and working memory in prodromal DLB patients. Prodromal DLB exhibited worse performance in memory and language than healthy controls, while showing better memory but worse performance in visual perception, attention, and executive tasks than MCI‐AD. Comparisons in some articles with MCI of other aetiologies confirmed the distinctiveness for prodromal DLB of this pattern of impairment. The most used tests to detect prodromal DLB included the Frontal Assessment Battery (FAB), Digit Symbol Substitution Test (DSST), Stroop test, AX continuous performance test (AX‐CPT), digit span backward, Rey‐Osterrieth Complex Figure copy, pareidolia test, and Visual Object and Space Perception battery (VOSP).

**Conclusions:**

Prodromal DLB can be detected using test batteries focused on specific cognitive domains and facilitating early diagnosis. It is possible to standardise a targeted test battery centred on specific visuoperceptual and attention domains for early detection of subtle cognitive decline due to Lewy body pathology.

## 1 Introduction

Dementia with Lewy bodies (DLB) is a prevalent neurodegenerative dementia [1] that predominantly affects individuals over 65 years of age [2]. It manifests as a spectrum, with an initial ‘prodromal’ or pre‐dementia phase that may present as mild cognitive impairment due to Lewy bodies (MCI‐LB) characterised by mild, yet distinctive, symptoms that do not compromise individual functional independence. It can also manifest, however, through other early clinical features such as prolonged and/or recurrent delirium, as manifestations of severe fluctuations of attention or a spectrum of psychiatric symptoms (anxiety‐depression‐apathy‐psychosis). Typical‐MCI‐ or atypical presentations may be associated with mild parkinsonism (bradykinesia, rest tremor or rigidity), cognitive fluctuations, REM‐sleep behaviour disorder (RBD) and, rarely, visual hallucinations [3]. DLB is part of the Lewy Body Disease (LBD) spectrum, encompassing Parkinson's Disease (PD) and Parkinson's Disease Dementia (PDD), conditions that share similar neuropathological progression [4]. LBD neuropathology is characterised by Lewy Body aggregation, primarily consisting of α‐Synuclein (α‐SYN), along with other proteins such as Tau, α‐Crystallin, and Ubiquitin, affecting the brainstem, limbic system, and neocortex. DLB is characterised by a significant accumulation of Lewy bodies, and subsequent neuronal loss, in the brainstem nuclei, particularly in the substantia nigra, paralimbic structures, neocortical areas, the locus coeruleus, and the olfactory nerve [1, 5]. LB accumulation results in neuronal death, neural pathways degeneration, primarily the dopaminergic ones, and gradual symptoms manifestation [6]. Additionally, degeneration of cholinergic connections in the brainstem and forebrain is frequently observed and leads to a substantial depletion of cortical cholinergic neurotransmitters [7].

Clinical diagnostic criteria for DLB and prodromal DLB have been proposed [3, 5], emphasising core clinical symptoms—cognitive fluctuations, RBD, parkinsonism, visual hallucinations—and early‐stage impairments, alongside potential biomarkers for the identification of the disease [3, 5]. Notably, while the criteria for DLB are intended for clinical use, the criteria for prodromal DLB (i.e., MCI‐LB) are currently designated for research purposes only. Other supportive features include hypersensitivity to antipsychotic agents, postural instability, repeated falls, syncope or transient episodes of unresponsiveness, severe autonomic dysfunction (constipation, orthostatic hypotension, urinary incontinence), hypersomnia, hyposmia, hallucinations in other sensory modalities, delusions, and mood disorders (anxiety, depression, apathy). For the prodromal DLB stage, three different phenotypical presentations have been proposed: (1) non‐amnestic MCI, defined by mild cognitive symptoms while memory and functional independence remain preserved, (2) delirium‐onset, characterised by prolonged or recurrent fluctuations in cognitive ability or function, (3) psychiatric‐onset, marked by new‐onset or atypical psychiatric symptoms such as hallucinations, delusions, mood or behavioural changes [3]. It is important to note that, although these represent prototypical syndromes of prodromal DLB, the appearance of other symptoms suggestive of DLB cannot be excluded at this stage [8]. Diagnosis of prodromal DLB may be categorised as probable or possible based on symptomatology and biomarker positivity. Indeed, research criteria for prodromal DLB [3, 5] rely on the use of various biomarkers. The main ones include reduced dopamine transporter uptake in basal ganglia (demonstrated through single photon emission computed tomography [SPECT] and positron emission tomography [PET]), reduced myocardial sympathetic functioning (demonstrated through myocardial scintigraphy), and confirmation of RBD without atonia (through polysomnography). Potential supportive biomarkers for prodromal DLB have also been proposed and consist of electroencephalography (EEG), and structural and functional magnetic resonance imaging (MRI) findings. However, to date, *post‐mortem* analysis remains the definitive diagnostic method to demonstrate LB presence, although research is exploring correlations between LB presence in the central nervous system (specifically α‐SYN) and its detection in biological fluids or peripheral tissues [9]. Although biofluid biomarkers identification will be crucial for an in vivo DLB diagnosis, it might require invasive procedures and high costs and therefore may not be scalable in primary care settings or general dementia clinics. Thus, it would be clinically helpful to identify clinical and neuropsychological features that can detect these patients in the earliest disease stage with a high level of sensitivity and specificity.

The neuropsychological profile of early Lewy body disease is characterised by attentional, executive, and visuospatial impairments, whereas memory tends to be relatively preserved compared with the prodromal stage of other neurodegenerative dementias, such as MCI due to Alzheimer's Disease (MCI‐AD) [3]. In contrast, patients with MCI due to Parkinson's Disease (MCI‐PD) differ from prodromal DLB individuals in the temporal onset of motor and cognitive symptoms—as defined by the ‘1‐year rule’, whereby motor symptoms should precede cognitive impairment by at least one year [3]—and in visuospatial deficits, typical of prodromal DLB but less pronounced in MCI‐PD [10, 11]. Thus, neuropsychological assessment is critical for characterising deficits in these cognitive domains, aiding subsequent neurological examinations. Greater research effort should be directed towards clarifying the cognitive impairments detectable in the prodromal phase to identify the initial symptoms of this condition as early as possible, thus facilitating patient management, treatment, and, potentially in the future, prevention of progression to dementia.

Although DLB has been the subject of numerous studies, the earliest altered cognitive domains are still being defined. A recent review and meta‐analysis represents a step in the right direction [12]. This study identified a neuropsychological profile of prodromal DLB consistent with the established profile of DLB with deficits in attention, processing speed and executive functions. However, this review focused on comparisons with DLB and MCI‐AD only, and included in its selection criteria patients with isolated RBD. Although RBD might be indicative of future DLB, a large proportion of patients with this disorder might never develop DLB [13, 14]. Thus, the findings of this study might not exclusively reflect the neuropsychological profile of prodromal DLB and their specificity remains limited. To overcome limitations and expand current knowledge, this systematic review aimed to identify the specific neuropsychological impairments that characterise the cognitive profile of prodromal DLB and the most suitable tests for detecting these impairments. Specifically, the objectives were to determine which neuropsychological tests are most commonly used in clinical practice worldwide to assess cognitive symptoms in the prodromal phase of DLB, to evaluate the potential utility of less commonly used tests in the early identification of this disease, and to examine whether comparisons between prodromal DLB patients and other populations (e.g., healthy controls or individuals with other neurodegenerative conditions) can support the definition of a specific neuropsychological profile for prodromal DLB. Ultimately, by reviewing studies that have investigated the neuropsychological profile of this stage of disease, this review aimed to define a battery of tests that can lead to accurate profiling of the earliest neuropsychological alterations detectable in patients who are at the prodromal stage of DLB.

## 2 Methods

### 2.1 Search Strategy

The review was conducted searching articles in the PubMed and Web of Science databases via specific keyword strings. Keywords used were centred on the scientific aim of the study, therefore coding the population of interest as ‘prodromal DLB/LBD/LB’, ‘prodromal Lewy bod*’ or ‘prodromal dementia with Lewy bod*’ but also ‘Mild cognitive impairment LB/LBD/DLB’ or ‘MCI‐LB/DLB/LBD’, while the neuropsychological evaluation was coded as ‘Neuropsycholog*’, ‘Cognitive test*’ or ‘Cognitive profil*’. Strings were linked through Boolean operators and their inclusion was restricted to the title and abstract of the articles, with the aim of combining accuracy and exclusivity from the early stages of selection. The search was conducted up to and including January 2025. See Table 1 for complete word strings.

**TABLE 1:** Keywords strings.

Database	Strings
PubMed	((‘neuropsycholog*’[Title/Abstract] OR ‘cognitive test’[Title/Abstract] OR ‘cognitive profil*’[Title/Abstract]) AND ‘prodromal dlb’[Title/Abstract]) OR ‘prodromal lewy bod*’[Title/Abstract] OR ‘prodromal dementia with lewy bodies’[Title/Abstract]
	(((((((((‘neuropsycholog*’[Title/Abstract] OR ‘cognitive test’[Title/Abstract] OR ‘cognitive profil*’[Title/Abstract])) AND (prodromal LB[Title/Abstract])) OR (prodromal LBD[Title/Abstract])) OR (MCI‐LB[Title/Abstract])) OR (MCI‐LBD[Title/Abstract])) OR (MCI‐DLB[Title/Abstract])) OR (mild cognitive impairment LB[Title/Abstract])) OR (mild cognitive impairment DLB[Title/Abstract])) OR (mild cognitive impairment LBD[Title/Abstract])
Web of Science	(((((TS = (neuropsycholog*)) OR TS = (cognitive profil*)) OR TS = (cognitive test)) AND TS = (prodromal DLB)) OR TS = (prodromal dementia with lewy bodies)) OR TS = (prodromal lewy bod*)
	((((((((((TS = (neuropsycholog*)) OR TS = (cognitive profil*)) OR TS = (cognitive test)) AND TS = (prodromal LBD)) OR TS = (MCI‐LB)) OR TS = (MCI‐DLB)) OR TS = (MCI‐LBD)) OR TS = (mild cognitive impairment LB)) OR TS = (mild cognitive impairment DLB)) OR TS = (mild cognitive impairment LBD)) OR TS = (prodromal LB)

### 2.2 Eligibility Criteria

The inclusion criteria for this systematic review were as follows: (1) original studies, (2) written in English, (3) peer‐reviewed articles, (4) studies assessing prodromal DLB populations or DLB patients including those in prodromal or MCI stages, and (5) evaluating at least one cognitive domain in depth other than a measure of global cognition. Papers were excluded if they met the following criteria: (1) reviews, case reports, meta‐analyses, editorial material or case series, (2) focused on populations with idiopathic RBD (iRBD), (3) evaluated only patients in prodromal or MCI stages of other neurodegenerative diseases, (4) evaluated patients solely through measures of global cognition (e.g., Mini‐Mental State Examination, Montreal Cognitive Assessment, Addenbrooke's Cognitive Examination), and (5) did not differentiate MCI of different aetiologies.

### 2.3 Study Selection

The study selection process comprised three distinct phases. Initially, all papers underwent a screening based on their title, aiming to remove duplicates and articles not directly pertinent to the research question. Subsequently, a comprehensive screening of abstracts was conducted, followed by the examination of full texts. Studies had to satisfy the predefined inclusion criteria at each step of the selection process. One researcher (F.M.) undertook screening, and another (G.Z.) joined for abstract and full‐text screening to ensure accuracy and consistency. In instances of discordance or ambiguous cases, intervention from other researchers (F.D.A., D.C.) was sought. Once verified the methods of all relevant abstracts to ascertain adherence to inclusion and exclusion criteria, rigorous analysis of full articles was undertaken, and the final screening was completed. Throughout the title and abstract screening phases, extraction of relevant metadata including first author, source title, DOI, publication year, and article title was conducted. Additionally, for pertinent abstracts and full‐text articles, comprehensive data inclusive of target populations, control groups (Healthy controls, HC), comparison populations (MCI‐AD, MCI due to other aetiologies), sample size, and detailed descriptions of the neuropsychological assessment administered were recorded.

## 3 Results

### 3.1 Selected Studies

**FIGURE 1:**
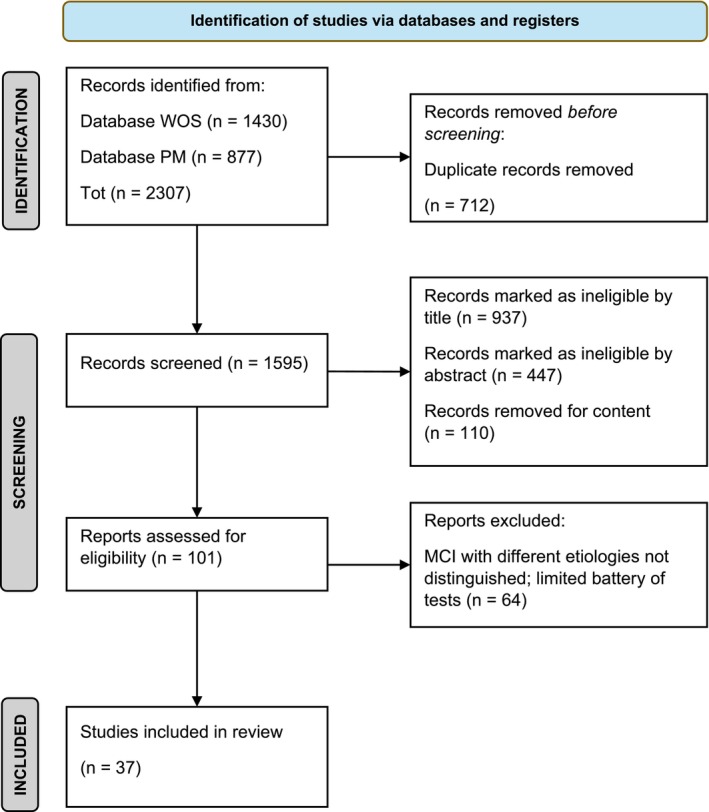
Flow chart detailing the article selection process.

Searches conducted through PubMed and Web of Science yielded a total of 2307 publications, with 1430 sourced from Web of Science and 877 from PubMed. Among these, 712 duplicates were identified and subsequently removed. The remaining articles underwent thorough title and abstract screening, resulting in the exclusion of 937 items based on title and an additional 447 items based on abstract content. Furthermore, 110 publications were identified as reviews, case reports, or meta‐analyses and were consequently excluded. Following these selection steps, a total of 101 studies remained. During the final screening phase, an additional 64 studies were deemed ineligible based on exclusion criteria, leaving a final selection of 37 papers upon which the review was conducted. The complete selection process is shown in Figure 1.

### 3.2 Neuropsychological Evaluation

To achieve the goals of this review, we counted the frequency of use of each test in all included studies and then combined the results of individual studies to reach an overall assessment of patients' cognitive impairments and performance on tests, differentiating prodromal DLB populations from HC and comparison populations (see Table 2 for a complete analysis).

**TABLE 2:** Summary table of the reviewed studies subdivided by cognitive domain.

Cognitive domain		NPS test	Target population	Other populations	Relevant results	Authors
Memory	Short term memory	Digit Span forward	9 VLOSLP + LB	20 VLOSLP‐LB	VLOSLP + LB = VLOSLP‐ LB	Kanemoto et al. (2022)
			25 pro‐DLB	28 MCI‐AD	Pro‐DLB = MCI‐AD	Cagnin et al. (2015A)
			23 pro‐DLB	36 HC 32 MCI‐AD	Pro‐DLB = MCI‐AD pro‐DLB = HC	Kim et al. (2018)
			30 pro‐DLB	23 MCI‐AD	Pro‐DLB = MCI‐AD	Cagnin et al. (2015B)
			44 prob. pro‐DLB	34 HC 39 MCI‐AD	Pro‐DLB = MCI‐AD Pro‐DLB = HC	Ciafone et al. (2022)
			18 pro‐DLB	32 MCI‐AD 45 MCI stable	Pro‐DLB = MCI‐AD	Yoon et al. (2015)
			73 pro‐DLB	124 MCI‐AD	Pro‐DLB < MCI‐AD	Van De Beek et al. (2020)
			15 poss pro‐DLB 39 prob. pro‐DLB	31 HC 35 MCI‐AD		Hamilton et al. (2023)
			19 pro‐DLB	25 MCI‐AD	Pro‐DLB = MCI‐AD	Bussè et al. (2018)
			20 pro‐DLB	46 MCI‐PD	Pro‐DLB = MCI‐PD	Yoon et al. (2014)
			74 pro‐DLB	1294 MCI‐AD	Pro‐DLB = MCI‐AD	Payne et al. (2022)
			15 pro‐DLB	13 HC 40 DLB	Pro‐DLB = HC Pro‐DLB = DLB	Jeong et al. (2024)
			143 pro‐DLB	429 MCI‐AD	Pro‐DLB = MCI‐AD	Wyman‐Chick et al. (2025)
		Corsi span forward	44 prob. pro‐DLB	34 HC 39 MCI‐AD	Pro‐DLB = MCI‐AD	Ciafone et al. (2022)
			15 poss pro‐DLB 39 prob. pro‐DLB	31 HC 35 MCI‐AD		Hamilton et al. (2023)
	Long term memory	Rey Auditory Verbal Learning test (Free recall)	28 prob. pro‐DLB	30 MCI‐AD	Pro‐DLB > MCI‐AD	Donaghy et al. (2022)
			44 prob. pro‐DLB	34 HC 39 MCI‐AD	Pro‐DLB = MCI‐AD	Ciafone et al. (2022)
			21 pro‐DLB	107 MCI‐AD 164 MCI stable	Pro‐DLB = MCI	Sadiq et al. (2017)
			73 pro‐DLB	124 MCI‐AD	Pro‐DLB > MCI‐AD	Van De Beek et al. (2020)
			15 poss pro‐DLB 39 prob. pro‐DLB	31 HC 35 MCI‐AD		Hamilton et al. (2023)
		Rey Auditory Verbal Learning test (delayed recall)	12 poss pro‐DLB 41 prob. pro‐DLB	23 MCI‐AD	Pro‐DLB = MCI‐AD	Hamilton et al. (2021B)
			28 prob. pro‐DLB	30 MCI‐AD	Pro‐DLB > MCI‐AD	Donaghy et al. (2022)
			41 pro‐DLB	24 MCI‐AD	Pro‐DLB = MCI‐AD	Donaghy et al. (2018)
			44 prob. pro‐DLB	34 HC 39 MCI‐AD	Pro‐DLB > MCI‐AD	Ciafone et al. (2022)
			49 pro‐DLB	162 MCI‐AD 116 MCI stable	Pro‐DLB > MCI‐AD	Ferman et al. (2013)
			21 pro‐DLB	107 MCI‐AD 164 MCI stable	Pro‐DLB > MCI‐AD	Sadiq et al. (2017)
			15 poss pro‐DLB 39 prob. pro‐DLB	31 HC 35 MCI‐AD		Hamilton et al. (2023)
		Rey Auditory Verbal Learning test (Recognition)	12 poss pro‐DLB 41 prob. pro‐DLB	23 MCI‐AD	Pro‐DLB = MCI‐AD	Hamilton et al. (2021B)
			28 prob. pro‐DLB	30 MCI‐AD	Pro‐DLB = MCI‐AD	Donaghy et al. (2022)
			41 pro‐DLB	24 MCI‐AD	Pro‐DLB = MCI‐AD	Donaghy et al. (2018)
		Seoul verbal learning test (Free recall)	23 poss pro‐DLB	36 HC 32 MCI‐AD	Pro‐DLB = MCI‐AD pro‐DLB = HC	Kim et al. (2018)
			18 pro‐DLB	32 MCI‐AD 45 MCI stable	Pro‐DLB = MCI‐AD Pro‐DLB < MCI stable	Yoon et al. (2015)
			20 pro‐DLB	46 MCI‐PD	Pro‐DLB = MCI‐PD	Yoon et al. (2014)
			15 pro‐DLB	13 HC 40 DLB	Pro‐DLB < HC	Jeong et al. (2024)
		Seoul verbal learning test (delayed recall)	23 poss pro‐DLB	36 HC	Pro‐DLB = MCI‐AD	Kim et al. (2018)
				32 MCI‐AD	Pro‐DLB = HC	
			18 pro‐DLB	32 MCI‐AD 45 MCI stable	Pro‐DLB = MCI‐AD Pro‐DLB < MCI stable	Yoon et al. (2015)
			20 pro‐DLB	46 MCI‐PD	Pro‐DLB < MCI‐PD	Yoon et al. (2014)
			15 pro‐DLB	13 HC 40 DLB	Pro‐DLB < HC	Jeong et al. (2024)
		Seoul verbal learning test (Recognition)	23 poss pro‐DLB	36 HC 32 MCI‐AD	Pro‐DLB = MCI‐AD pro‐DLB = HC	Kim et al. (2018)
			18 pro‐DLB	32 MCI‐AD 45 MCI stable	Pro‐DLB = MCI‐AD Pro‐DLB < MCI stable	Yoon et al. (2015)
			20 pro‐DLB	46 MCI‐PD	Pro‐DLB < MCI‐PD	Yoon et al. (2014)
			15 pro‐DLB	13 HC 40 DLB	Pro‐DLB < HC	Jeong et al. (2024)
		Hopkins verbal learning task (immediate)	14 pro‐DLB	57 MCI‐AD 53 DLB 100 AD 5 VaD 10 FTD 9 other	Pro‐DLB = MCI‐AD	Galvin (2015)
		Hopkins verbal learning task (delayed)	14 pro‐DLB	57 MCI‐AD 53 DLB 100 AD 5 VaD 10 FTD 9 other	Pro‐DLB = MCI‐AD	Galvin (2015)
		Hopkins verbal learning task (cued)	14 pro‐DLB	57 MCI‐AD 53 DLB 100 AD 5 VaD 10 FTD 9 other	Pro‐DLB = MCI‐AD	Galvin (2015)
		Philadelphia verbal learning test	38 pro‐DLB	59 HC	Pro‐DLB < HC	Železníková et al. (2024)
			35 MCI‐DLB	28 HC	Pro‐DLB < HC	Novakova et al. (2024)
		FC‐SRT (RL‐RI 16)	26 prob. DLB + pro‐DLB	19 HC	DLB = HC	Falque et al. (2022)
			148 pro‐DLB	469 no symptom 275 1 symptom	Pro‐DLB = no symptom	Blanc et al. (2022)
			19 pro‐DLB	25 MCI‐AD	Pro‐DLB > MCI‐AD	Bussè et al. (2018)
			91 DLB (pro‐ DLB + mild DLB)	18 HC 28 AD 15 DLB + AD	DLB > AD DLB < HC DLB < DLB + AD	Querry et al. (2023)
		CERAD word list	9 pro‐DLB	12 MCI‐AD	Pro‐DLB = MCI‐AD	Jicha et al. (2010)
		Story recall (free recall)	25 pro‐DLB	28 MCI‐AD	Pro‐DLB = MCI‐AD	Cagnin et al. (2015A)
			30 pro‐DLB	23 MCI‐AD	Pro‐DLB > MCI‐AD	Cagnin et al. (2015B)
			19 pro‐DLB	25 MCI‐AD	Pro‐DLB = MCI‐AD	Bussè et al. (2018)
		Story recall (delayed recall)	25 pro‐DLB	28 MCI‐AD	Pro‐DLB > MCI‐AD	Cagnin et al. (2015A)
			30 pro‐DLB	23 MCI‐AD	Pro‐DLB > MCI‐AD	Cagnin et al. (2015B)
			19 pro‐DLB	25 MCI‐AD	Pro‐DLB = MCI‐AD	Bussè et al. (2018)
		Wechsler memory scale revised: Logical memory I A	14 pro‐DLB	46 HC 38 MCI‐AD	Pro‐DLB = MCI‐AD pro‐DLB < HC	Kobayashi et al. (2023)
			27 DLB (DLB + pro‐ DLB)	47 AD (AD + MCI‐ AD) 49 HC	DLB > AD DLB < HC	Yamada et al. (2022)
			111 pro‐DLB	501 MCI‐AD	Pro‐DLB > MCI‐AD	Ting et al. (2023)
			74 pro‐DLB	1278 MCI‐AD	Pro‐DLB = MCI‐AD	Payne et al. (2022)
			9 pro‐DLB	12 MCI‐AD	Pro‐DLB > MCI‐AD	Jicha et al. (2010)
			21 pro‐DLB	107 MCI‐AD 164 MCI stable	Pro‐DLB = MCI	Sadiq et al. (2017)
			11 VLOSLP + LB	23 VLOSLP‐LB	VLOSLP + LB = VLOSLP‐LB	Kanemoto H et al. (2022)
			143 pro‐DLB	429 MCI‐AD	Pro‐DLB > MCI‐AD	Wyman‐Chick et al. (2025)
			Pro‐DLB	MCI‐AD MCI‐VaD	Pro‐DLB > MCI‐AD Pro‐DLB = MCI‐FTD	Liampas et al. (2024)
				MCI‐FTD HC	Pro‐DLB = MCI‐VaD Pro‐DLB < HC	
		Wechsler memory scale revised: Logical memory II A	14 pro‐DLB	46 HC 38 MCI‐AD	Pro‐DLB > MCI‐AD pro‐DLB < HC	Kobayashi et al. (2023)
			27 DLB (DLB + pro‐ DLB)	47 AD (AD + MCI‐ AD) 49 HC	DLB > AD DLB < HC	Yamada et al. (2022)
			111 pro‐DLB	501 MCI‐AD	Pro‐DLB > MCI‐AD	Ting et al. (2023)
			49 pro‐DLB	162 MCI‐AD 116 MCI stable	Pro‐DLB > MCI‐AD	Ferman et al. (2013)
			21 pro‐DLB	107 MCI‐AD 164 MCI stable	Pro‐DLB > MCI‐AD	Sadiq et al. (2017)
			74 pro‐DLB	1278 MCI‐AD	Pro‐DLB > MCI‐AD	Payne et al. (2022)
			11 VLOSLP + LB	23 VLOSLP‐LB	VLOSLP + LB = VLOSLP‐ LB	Kanemoto H et al. (2022)
		Rey complex figure (immediate recall)	23 poss pro‐DLB	36 HC 32 MCI‐AD	Pro‐DLB = MCI‐AD Pro‐DLB < HC	Kim et al. (2018)
			18 pro‐DLB	32 MCI‐AD 45 MCI stable	Pro‐DLB = MCI‐AD Pro‐DLB < MCI stable	Yoon et al. (2015)
			20 pro‐DLB	46 MCI‐PD	Pro‐DLB < MCI‐PD	Yoon et al. (2014)
			15 pro‐DLB	13 HC 40 DLB	Pro‐DLB < HC	Jeong et al. (2024)
		Rey complex figure (delayed recall)	23 poss pro‐DLB	36 HC 32 MCI‐AD	Pro‐DLB = MCI‐AD Pro‐DLB < HC	Kim et al. (2018)
			25 pro‐DLB	28 MCI‐AD	Pro‐DLB = MCI‐AD	Cagnin et al. (2015A)
			30 pro‐DLB	23 MCI‐AD	Pro‐DLB = MCI‐AD	Cagnin et al. (2015B)
			18 pro‐DLB	32 MCI‐AD 45 MCI stable	Pro‐DLB = MCI‐AD Pro‐DLB < MCI stable	Yoon et al. (2015)
			148 pro‐DLB	469 no symptom 275 1 symptom	Pro‐DLB = no symptom	Blanc et al. (2022)
			19 pro‐DLB	25 MCI‐AD	Pro‐DLB = MCI‐AD	Bussè et al. (2018)
			20 pro‐DLB	46 MCI‐PD	Pro‐DLB < MCI‐PD	Yoon et al. (2014)
			15 pro‐DLB	13 HC 40 DLB	Pro‐DLB < HC Pro‐DLB > DLB	Jeong et al. (2024)
		Taylor complex figure (immediate recall)	44 prob. pro‐DLB	34 HC 39 MCI‐AD	Pro‐DLB = MCI‐AD	Ciafone et al. (2022)
		Taylor complex figure (delayed recall)	44 prob. pro‐DLB	34 HC 39 MCI‐AD	Pro‐DLB > MCI‐AD	Ciafone et al. (2022)
		Benson complex figure (immediate recall)	143 pro‐DLB	429 MCI‐AD	Pro‐DLB = MCI‐AD	Wyman‐Chick et al. (2025)
		Benson complex figure (delayed recall)	143 pro‐DLB	429 MCI‐AD	Pro‐DLB > MCI‐AD	Wyman‐Chick et al. (2025)
		Visual memory (DMS48)	91 DLB (pro‐DLB + mild DLB)	18 HC 28 AD 15 DLB + AD	DLB > AD DLB < HC DLB = DLB + AD	Querry et al. (2023)
		Visuospatial memory test revised	38 pro‐DLB	59 HC	Pro‐DLB < HC	Železníková et al. (2024)
			35 MCI‐DLB	28 HC	Pro‐DLB < HC	Novakova et al. (2024)
		Visual association test	63 pro‐DLB	124 MCI‐AD	Pro‐DLB > MCI‐AD	Van De Beek et al. (2020)
Working memory		Digit span backward	75 DLB (pro‐DLB + DLB)	91 HC 121 AD + VaD 140 MCI	DLB < controls	Garcia Basalo et al. (2017)
			9 VLOSLP + LB	20 VLOSLP‐LB	VLOSLP + LB = VLOSLP‐LB	Kanemoto H et al. (2022)
			23 poss pro‐DLB	36 HC 32 MCI‐AD	Pro‐DLB = MCI‐AD pro‐DLB = HC	Kim et al. (2018)
			30 pro‐DLB	23 MCI‐AD	Pro‐DLB < MCI‐AD	Cagnin et al. (2015B)
			44 prob. pro‐DLB	34 HC 39 MCI‐AD	Pro‐DLB = MCI‐AD	Ciafone et al. (2022)
			18 pro‐DLB	32 MCI‐AD 45 MCI stable	Pro‐DLB = MCI‐AD	Yoon et al. (2015)
			25 pro‐DLB	28 MCI‐AD	Pro‐DLB < MCI‐AD	Cagnin et al. (2015A)
			73 pro‐DLB	124 MCI‐AD	Pro‐DLB < MCI‐AD	Van De Beek et al. (2020)
			15 poss pro‐DLB 39 prob. pro‐DLB	31 HC 35 MCI‐AD		Hamilton et al. (2023)
			19 pro‐DLB	25 MCI‐AD	Pro‐DLB = MCI‐AD	Bussè et al. (2018)
			20 pro‐DLB	46 MCI‐PD	Pro‐DLB = MCI‐PD	Yoon et al. (2014)
			74 pro‐DLB	1294 MCI‐AD	Pro‐DLB < MCI‐AD	Payne et al. (2022)
			15 pro‐DLB	13 HC 40 DLB	Pro‐DLB < HC	Jeong et al. (2024)
			143 pro‐DLB	429 MCI‐AD	Pro‐DLB < MCI‐AD	Wyman‐Chick et al. (2025)
		Corsi span backward	44 prob. pro‐DLB	34 HC 39 MCI‐AD	Pro‐DLB = MCI‐AD	Ciafone et al. (2022)
			15 poss pro‐DLB 39 prob. pro‐DLB	31 HC 35 MCI‐AD		Hamilton et al. (2023)
		Letter number sequencing (WAIS III)	14 pro‐DLB	57 MCI‐AD 53 DLB 100 AD 5 VaD 10 FTD 9 other	Pro‐DLB = MCI‐AD	Galvin (2015 (2))
			38 pro‐DLB	59 HC	Pro‐DLB < HC	Železníková et al. (2024)
			35 MCI‐DLB	28 HC	Pro‐DLB < HC	Novakova et al. (2024)
Visual‐perceptive/Visual‐spatial/Visual‐constructive	Visual‐perceptual abilities	VOSP Visual perceptual tasks	25 pro‐DLB	28 MCI‐AD	Pro‐DLB = MCI‐AD	Cagnin et al. (2015A)
			148 pro‐DLB	469 no symptom 275 1 symptom	Pro‐DLB < no symptom	Blanc et al. (2022)
			73 pro‐DLB	124 MCI‐AD	Pro‐DLB < MCI‐AD	Van De Beek et al. (2020)
		Pareidolia test (Total)	22 pro‐DLB	70 AD 110 DLB 79 MCI‐AD	Pro‐DLB < MCI‐AD	Galvin et al. (2021)
			20 poss pro‐DLB 43 prob. pro‐DLB	34 HC 40 MCI‐AD	Pro‐DLB < MCI‐AD Pro‐DLB < HC	Hamilton et al. (2021 A)
	Visual‐spatial abilities	Judgement of line orientation	49 pro‐DLB	162 MCI‐AD 116 MCI stable	Pro‐DLB < MCI‐AD	Ferman et al. (2013)
			38 pro‐DLB	59 HC	Pro‐DLB < HC	Železníková et al. (2024)
			35 MCI‐DLB	28 HC	Pro‐DLB < HC	Novakova et al. (2024)
		Line angle discrimination	28 prob. pro‐DLB	30 MCI‐AD	Pro‐DLB = MCI‐AD	Donaghy et al. (2022)
			41 pro‐DLB	24 MCI‐AD	Pro‐DLB < MCI‐AD	Donaghy et al. (2018)
			15 poss pro‐DLB 39 prob. pro‐DLB	31 HC 35 MCI‐AD		Hamilton et al. (2023)
			12 poss pro‐DLB 41 prob. pro‐DLB	23 MCI‐AD	Prob pro‐LB < poss pro‐LB	Hamilton et al. (2021B)
		VOSP Visual spatial tasks	25 pro‐DLB	28 MCI‐AD	Pro‐DLB < MCI‐AD	Cagnin et al. (2015A)
			148 pro‐DLB	469 no symptom 275 1 symptom	Pro‐DLB < no symptom	Blanc et al. (2022)
			73 pro‐DLB	124 MCI‐AD	Pro‐DLB < MCI‐AD	Van De Beek et al. (2020)
	Visual‐constructive abilities	Rey complex figure (copy)	25 pro‐DLB	28 MCI‐AD	Pro‐DLB < MCI‐AD	Cagnin et al. (2015A)
			23 poss pro‐DLB	36 HC 32 MCI‐AD	Pro‐DLB < HC Pro‐DLB = MCI‐AD	Kim et al. (2018)
			30 pro‐DLB	23 MCI‐AD	Pro‐DLB = MCI‐AD	Cagnin et al. (2015B)
			49 pro‐DLB	162 MCI‐AD 116 MCI stable	Pro‐DLB < MCI‐AD	Ferman et al. (2013)
			18 pro‐DLB	32 MCI‐AD 45 MCI stable	Pro‐DLB < MCI‐AD Pro‐DLB < MCI stable	Yoon et al. (2015)
			20 pro‐DLB	46 MCI‐PD	Pro‐DLB < MCI‐PD	Yoon et al. (2014)
			19 pro‐DLB	25 MCI‐AD	Pro‐DLB = MCI‐AD	Bussè et al. (2018)
			15 pro‐DLB	13 HC	Pro‐DLB = HC	Jeong et al. (2024)
				40 DLB	Pro‐DLB > DLB	
		Taylor complex figure (copy)	44 prob. pro‐DLB	34 HC 39 MCI‐AD	Pro‐DLB = MCI‐AD	Ciafone et al. (2022)
			15 poss pro‐DLB 39 prob. pro‐DLB	31 HC 35 MCI‐AD		Hamilton et al. (2023)
		Benson figure (copy)	143 pro‐DLB	429 MCI‐AD	Pro‐DLB < MCI‐AD	Wyman‐Chick et al. (2025)
		Clock drawing test	75 DLB (PRO‐ DLB + DLB)	91 HC 121 AD + VaD 140 MCI	DLB < controls	Garcia Basalo et al. (2017)
			14 pro‐DLB	46 HC 38 MCI‐AD	Pro‐DLB = MCI‐AD pro‐DLB = HC	Kobayashi et al. (2023)
			27 DLB (DLB + pro‐ DLB)	47 AD (AD + MCI‐ AD) 49 HC	DLB = AD DLB = HC	Yamada et al. (2022)
			30 pro‐DLB	23 MCI‐AD	Pro‐DLB < MCI‐AD	Cagnin et al. (2015B)
			14 pro‐DLB	57 MCI‐AD 53 DLB 100 AD 5 VaD 10 FTD 9 other	Pro‐DLB = MCI‐AD	Galvin (2015 (2))
			67 poss pro‐DLB	56 MCI‐PD	Pro‐DLB = MCI‐PD	Wright et al. (2023)
			21 pro‐DLB	107 MCI‐AD 164 MCI stable	Pro‐DLB < MCI	Sadiq et al. (2017)
			19 pro‐DLB	25 MCI‐AD	Pro‐DLB = MCI‐AD	Bussè et al. (2018)
		MMSE: pentagons intersection copy	27 DLB (DLB + pro‐ DLB)	47 AD (AD + MCI‐ AD) 49 HC	DLB = AD DLB < HC	Yamada et al. (2022)
			25 pro‐DLB	28 MCI‐AD	Pro‐DLB = MCI‐AD	Cagnin et al. (2015A)
			30 pro‐DLB	23 MCI‐AD	Pro‐DLB = MCI‐AD	Cagnin et al. (2015B)
			75 DLB (PRO‐ DLB + DLB)	91 HC 121 AD + VaD 140 MCI	DLB < controls	Garcia Basalo et al. (2017)
			19 pro‐DLB	25 MCI‐AD	Pro‐DLB < MCI‐AD	Bussè et al. (2018)
		WAIS: Block design test	9 VLOSLP + LB	20 VLOSLP‐LB	VLOSLP + LB = VLOSLP‐LB	Kanemoto H et al. (2022)
			49 pro‐DLB	162 MCI‐AD 116 MCI stable	Pro‐DLB < MCI‐AD	Ferman et al. (2013)
Language	Production	Boston Naming test	111 pro‐DLB	501 MCI‐AD	Pro‐DLB > MCI‐AD	Ting et al. (2023)
			23 poss pro‐DLB	36 HC 32 MCI‐AD	Pro‐DLB = MCI‐AD pro‐DLB < HC	Kim et al. (2018)
			14 pro‐DLB	57 MCI‐AD 53 DLB 100 AD 5 VaD 10 FTD 9 other	Pro‐DLB = MCI‐AD	Galvin (2015)
			49 pro‐DLB	162 MCI‐AD 116 MCI stable	Pro‐DLB = MCI‐AD	Ferman et al. (2013)
			18 pro‐DLB	32 MCI‐AD 45 MCI stable	Pro‐DLB = MCI‐AD Pro‐DLB < MCI stable	Yoon et al. (2015)
			9 pro‐DLB	12 MCI‐AD	Pro‐DLB = MCI‐AD	Jicha et al. (2010)
			20 pro‐DLB	46 MCI‐PD	Pro‐DLB = MCI‐PD	Yoon et al. (2014)
			74 pro‐DLB	1288 MCI‐AD	Pro‐DLB = MCI‐AD	Payne et al. (2022)
			15 pro‐DLB	13 HC 40 DLB	Pro‐DLB = HC Pro‐DLB < DLB	Jeong et al. (2024)
			143 pro‐DLB	429 MCI‐AD	Pro‐DLB > MCI‐AD	Wyman‐Chick et al. (2025)
			Pro‐DLB	MCI‐AD MCI‐VaD MCI‐FTD HC	Pro‐DLB = MCI‐AD Pro‐DLB = MCI‐VaD Pro‐DLB > MCI‐FTD Pro‐DLB < HC	Liampas et al. (2024)
		Graded naming test	12 poss pro‐DLB 41 prob. pro‐DLB	23 MCI‐AD	Pro‐DLB = MCI‐AD	Hamilton et al. (2021B)
			28 prob. pro‐DLB	30 MCI‐AD	Pro‐DLB = MCI‐AD	Donaghy et al. (2022)
			41 pro‐DLB	24 MCI‐AD	Pro‐DLB = MCI‐AD	Donaghy et al. (2018)
			44 prob. pro‐DLB	34 HC 39 MCI‐AD	Pro‐DLB = MCI‐AD	Ciafone et al. (2022)
			15 poss pro‐DLB 39 prob. pro‐DLB	31 HC 35 MCI‐AD		Hamilton et al. (2023)
		DO80	26 prob. DLB + pro‐DLB	19 HC	DLB < HC	Falque et al. (2022)
		MINT	22 pro‐DLB	70 AD 110 DLB 79 MCI‐AD	Pro‐DLB = MCI‐AD	Galvin et al. (2021)
			143 pro‐DLB	429 MCI‐AD	Pro‐DLB > MCI‐AD	Wyman‐Chick et al. (2025)
	Comprehension	Syntactic comprension (GREMOTs)	26 prob. DLB + pro‐ DLB	19 HC	DLB = HC	Falque et al. (2022)
Attention		Digit vigilance task	12 poss pro‐DLB 41 prob. pro‐DLB	23 MCI‐AD	Pro‐DLB = MCI‐AD	Hamilton et al. (2021B)
			28 prob. pro‐DLB	30 MCI‐AD	Pro‐DLB = MCI‐AD	Donaghy et al. (2022)
			41 pro‐DLB	24 MCI‐AD	Pro‐DLB = MCI‐AD	Donaghy et al. (2018)
		Simple reaction task	12 poss pro‐DLB 41 prob. pro‐DLB	23 MCI‐AD	Pro‐DLB = MCI‐AD	Hamilton et al. (2021B)
			28 prob. pro‐DLB	30 MCI‐AD	Pro‐DLB = MCI‐AD	Donaghy et al. (2022)
			41 pro‐DLB	24 MCI‐AD	Pro‐DLB = MCI‐AD	Donaghy et al. (2018)
			44 prob. pro‐DLB	34 HC 39 MCI‐AD	Pro‐DLB = MCI‐AD Pro‐DLB = HC	Ciafone et al. (2022)
			64 poss pro‐DLB	55 MCI‐PD	Pro‐DLB = MCI‐PD	Wright et al. (2023)
		Choice reaction task	12 poss pro‐DLB 41 prob. pro‐DLB	23 MCI‐AD	Pro‐DLB = MCI‐AD	Hamilton et al. (2021 B)
			28 prob. pro‐DLB	30 MCI‐AD	Pro‐DLB = MCI‐AD	Donaghy et al. (2022)
			41 pro‐DLB	24 MCI‐AD	Pro‐DLB = MCI‐AD	Donaghy et al. (2018)
			64 poss pro‐DLB	55 MCI‐PD	Pro‐DLB > MCI‐PD	Wright et al. (2023)
			37 prob. pro‐DLB 12 poss pro‐DLB	50 HC	Pro‐DLB < HC	Revie et al. (2020)
		AX CPT	15 poss pro‐DLB 39 prob. pro‐DLB	31 HC 35 MCI‐AD	Pro‐DLB < MCI AD Pro‐DLB < HC	Hamilton et al. (2023)
		Trail making test A	14 pro‐DLB	46 HC 38 MCI‐AD	Pro‐DLB = MCI‐AD pro‐DLB < HC	Kobayashi et al. (2023)
			27 DLB (DLB + pro‐ DLB)	47 AD (AD + MCI‐ AD) 49 HC	DLB < HC DLB = AD	Yamada et al. (2022)
			22 pro‐DLB	70 AD 110 DLB 79 MCI‐AD	Pro‐DLB < MCI‐AD	Galvin et al. (2021)
			25 pro‐DLB	28 MCI‐AD	Pro‐DLB < MCI‐AD	Cagnin et al. (2015A)
			111 pro‐DLB	501 MCI‐AD	Pro‐DLB < MCI‐AD	Ting et al. (2023)
			12 poss pro‐DLB 41 prob. pro‐DLB	23 MCI‐AD	Pro‐DLB = MCI‐AD	Hamilton et al. (2021B)
			28 pro‐DLB	27 MCI‐AD 31 DLB 54 AD 33 HC	Pro‐DLB > DLB	Blanc et al. (2015)
			30 pro‐DLB	23 MCI‐AD	Pro‐DLB < MCI‐AD	Cagnin et al. (2015B)
			14 pro‐DLB	57 MCI‐AD 53 DLB 100 AD 5 VaD 10 FTD 9 other	Pro‐DLB = MCI‐AD	Galvin (2015)
			28 prob. pro‐DLB	30 MCI‐AD	Pro‐DLB = MCI‐AD	Donaghy et al. (2022)
			41 pro‐DLB	24 MCI‐AD	Pro‐DLB = MCI‐AD	Donaghy et al. (2018)
			93 DLB (pro to moderate)	28 HC	Pro‐DLB < HC	Botzung et al. (2019)
			148 pro‐DLB	469 no symptom 275 1 symptom	Pro‐DLB > no symptom	Blanc et al. (2022)
			9 pro‐DLB	12 MCI‐AD	Pro‐DLB < MCI‐AD	Jicha et al. (2010)
			21 pro‐DLB	107 MCI‐AD 164 MCI stable	Pro‐DLB < MCI	Sadiq et al. (2017)
			19 pro‐DLB	25 MCI‐AD	Pro‐DLB = MCI‐AD	Bussè et al. (2018)
			92 pro‐DLB	1439 MCI‐AD	Pro‐DLB < MCI‐AD	Payne et al. (2022)
			143 pro‐DLB	429 MCI‐AD	Pro‐DLB < MCI‐AD	Wyman‐Chick et al. (2025)
			Pro‐DLB	MCI‐AD MCI‐VaD MCI‐FTD HC	Pro‐DLB < MCI‐AD Pro‐DLB = MCI‐VaD Pro‐DLB < MCI‐FTD Pro‐DLB < HC	Liampas et al. (2024)
		Digit cancellation (Letter cancellation)	25 pro‐DLB	28 MCI‐AD	Pro‐DLB = MCI‐AD	Cagnin et al. (2015A)
			30 pro‐DLB	23 MCI‐AD	Pro‐DLB = MCI‐AD	Cagnin et al. (2015B)
			19 pro‐DLB	25 MCI‐AD	Pro‐DLB < MCI‐AD	Bussè et al. (2018)
			20 pro‐DLB	46 MCI‐PD	Pro‐DLB = MCI‐PD	Yoon et al. (2014)
Executive‐ Attentional		Trail making test B	14 pro‐DLB	46 HC 38 MCI‐AD	Pro‐DLB < MCI‐AD pro‐DLB < HC	Kobayashi et al. (2023)
			27 DLB (DLB + pro‐ DLB)	47 AD (AD + MCI‐ AD) 49 HC	DLB < HC DLB = AD	Yamada et al. (2022)
			22 pro‐DLB	70 AD 110 DLB	Pro‐DLB < MCI‐AD	Galvin et al. (2021)
				79 MCI‐AD		
			111 pro‐DLB	501 MCI‐AD	Pro‐DLB < MCI‐AD	Ting et al. (2023)
			12 poss pro‐DLB 41 prob. pro‐DLB	23 MCI‐AD	Pro‐DLB = MCI‐AD	Hamilton et al. (2021 B)
			28 pro‐DLB	27 MCI‐AD 31 DLB 54 AD 33 HC	Pro‐DLB < HC	Blanc et al. (2015)
			14 pro‐DLB	57 MCI‐AD 53 DLB 100 AD 5 VaD 10 FTD 9 other	Pro‐DLB = MCI‐AD	Galvin (2015)
			28 prob. pro‐DLB	30 MCI‐AD	Pro‐DLB = MCI‐AD	Donaghy et al. (2022)
			41 pro‐DLB	24 MCI‐AD	Pro‐DLB = MCI‐AD	Donaghy et al. (2018)
			44 prob. pro‐DLB	34 HC 39 MCI‐AD	Pro‐DLB < MCI‐AD	Ciafone et al. (2022)
			148 pro‐DLB	469 no symptom 275 1 symptom	Pro‐DLB = no symptom	Blanc et al. (2022)
			9 pro‐DLB	12 MCI‐AD	Pro‐DLB = MCI‐AD	Jicha et al. (2010)
			12 pro‐DLB	80 MCI‐AD 126 MCI stable	Pro‐DLB = MCI	Sadiq et al. (2017)
			92 pro‐DLB	1439 MCI‐AD	Pro‐DLB < MCI‐AD	Payne et al. (2022)
			49 pro‐DLB	162 MCI‐AD 116 MCI stable	Pro‐DLB < MCI‐AD	Ferman et al. (2013)
			143 pro‐DLB	429 MCI‐AD	Pro‐DLB < MCI‐AD	Wyman‐Chick et al. (2025)
			Pro‐DLB	MCI‐AD MCI‐VaD MCI‐FTD HC	Pro‐DLB < MCI‐AD Pro‐DLB < MCI‐VaD Pro‐DLB < MCI‐FTD Pro‐DLB < HC	Liampas et al. (2024)
		Stroop test	23 poss pro‐DLB	36 HC	Pro‐DLB < MCI‐AD	Kim et al. (2018)
				32 MCI‐AD		
			44 prob. pro‐DLB	34 HC 39 MCI‐AD	Pro‐DLB < MCI‐AD	Ciafone et al. (2022)
			49 pro‐DLB	162 MCI‐AD 116 MCI stable	Pro‐DLB < MCI‐AD	Ferman et al. (2013)
			18 pro‐DLB	32 MCI‐AD 45 MCI stable	Pro‐DLB < MCI‐AD Pro‐DLB < MCI stable	Yoon et al. (2015)
			73 pro‐DLB	124 AD	Ori‐DLB < MCI‐AD	Van De Beek et al. (2020)
			15 poss pro‐DLB 39 prob. pro‐DLB	31 HC 35 MCI‐AD		Hamilton et al. (2023)
			20 pro‐DLB	46 MCI‐PD	Pro‐DLB < MCI‐PD	Yoon et al. (2014)
			15 pro‐DLB	13 HC 40 DLB	Pro‐DLB < HC Pro‐DLB > DLB	Jeong et al. (2024)
		Digit symbol substitution test (DSST)	10 VLOSLP + LB	20 VLOSLP‐LB	VLOSLP + LB < VLOSLP‐LB	Kanemoto H et al. (2022)
			44 prob. pro‐DLB	34 HC 39 MCI‐AD	Pro‐DLB < MCI‐AD	Ciafone et al. (2022)
			93 DLB (pro to moderate)	28 HC	Pro‐DLB < HC	Botzung et al. (2019)
			15 poss pro‐DLB 39 prob. pro‐DLB	31 HC 35 MCI‐AD		Hamilton et al. (2023)
			143 pro‐DLB	429 MCI‐AD	Pro‐DLB < MCI‐AD	Wyman‐Chick et al. (2025)
			35 MCI‐DLB	28 HC	Pro‐DLB < HC	Novakova et al. (2024)
			38 pro‐DLB	59 HC	Pro‐DLB < HC	Železníková et al. (2024)
Executive functions		FAB	27 DLB (DLB + pro‐ DLB)	47 AD (AD + MCI‐ AD) 49 HC	DLB < HC DLB = AD	Yamada et al. (2022)
			26 prob. DLB + pro‐DLB	19 HC	DLB < HC	Falque et al. (2022)
			73 pro‐DLB	124 MCI‐AD	Pro‐DLB < MCI‐AD	Van De Beek et al. (2020)
		(Contrasting program task) (Go‐no‐go task)	18 pro‐DLB	32 MCI‐AD 45 MCI stable	Pro‐DLB < MCI‐AD	Yoon et al. (2015)
			20 pro‐DLB	46 MCI‐PD	Pro‐DLB = MCI‐PD	Yoon et al. (2014)
			20 pro‐DLB	46 MCI‐PD	Pro‐DLB < MCI‐PD	Yoon et al. (2014)
			18 pro‐DLB	32 MCI‐AD 45 MCI stable	Pro‐DLB < MCI‐AD	Yoon et al. (2015)
		Picture arrangement test	38 pro‐DLB	59 HC	Pro‐DLB < HC	Železníková et al. (2024)
			35 MCI‐DLB	28 HC	Pro‐DLB < HC	Novakova et al. (2024)
		Category verbal fluency	22 pro‐DLB	70 AD 110 DLB 79 MCI‐AD	Pro‐DLB = MCI‐AD	Galvin et al. (2021)
			14 pro‐DLB	57 MCI‐AD 53 DLB 100 AD 5 VaD 10 FTD 9 other	Pro‐DLB = MCI‐AD	Galvin (2015)
			49 pro‐DLB	162 MCI‐AD 116 MCI stable	Pro‐DLB = MCI‐AD	Ferman et al. (2013)
			67 poss pro‐DLB	56 MCI‐PD	Pro‐DLB = MCI‐PD	Wright et al. (2023)
			148 pro‐DLB	469 no symptom 275 1 symptom	Pro‐DLB < no symptom	Blanc et al. (2022)
			9 pro‐DLB	12 MCI‐AD	Pro‐DLB = MCI‐AD	Jicha et al. (2010)
			21 pro‐DLB	107 MCI‐AD 164 MCI stable	Pro‐DLB = MCI	Sadiq et al. (2017)
			73 pro‐DLB	124 MCI‐AD	Pro‐DLB < MCI‐AD	Van De Beek et al. (2020)
			19 pro‐DLB	25 MCI‐AD	Pro‐DLB = MCI‐AD	Bussè et al. (2018)
			94 pro‐DLB	1468 MCI‐AD	Pro‐DLB = MCI‐AD	Payne et al. (2022)
			18 pro‐DLB	32 MCI‐AD 45 MCI stable	Pro‐DLB = MCI‐AD Pro‐DLB < MCI stable	Yoon et al. (2015)
			20 pro‐DLB	46 MCI‐PD	Pro‐DLB = MCI‐PD	Yoon et al. (2014)
			15 pro‐DLB	13 HC 40 DLB	Pro‐DLB < HC	Jeong et al. (2024)
			143 pro‐DLB	429 MCI‐AD	Pro‐DLB = MCI‐AD	Wyman‐Chick et al. (2025)
			38 pro‐DLB	59 HC	Pro‐DLB < HC	Železníková et al. (2024)
			Pro‐DLB	MCI‐AD MCI‐VaD MCI‐FTD HC	Pro‐DLB = MCI‐AD Pro‐DLB = MCI‐VaD Pro‐DLB > MCI‐FTD Pro‐DLB < HC	Liampas et al. (2024)
			35 MCI‐DLB	28 HC	Pro‐DLB < HC	Novakova et al. (2024)
		Letter verbal fluency (COWAT) (FAS)	25 pro‐DLB	28 MCI‐AD	Pro‐DLB < MCI‐AD	Cagnin et al. (2015 A)
			12 poss pro‐DLB 41 prob. pro‐DLB	23 MCI‐AD	Pro‐DLB = MCI‐AD	Hamilton et al. (2021 B)
			23 poss pro‐DLB	36 HC 32 MCI‐AD	Pro‐DLB = MCI‐ AD = HC	Kim et al. (2018)
			30 pro‐DLB	23 MCI‐AD	Pro‐DLB = MCI‐AD	Cagnin et al. (2015B)
			28 prob. pro‐DLB	30 MCI‐AD	Pro‐DLB = MCI‐AD	Donaghy et al. (2022)
			41 pro‐DLB	24 MCI‐AD	Pro‐DLB = MCI‐AD	Donaghy et al. (2018)
			44 prob. pro‐DLB	34 HC 39 MCI‐AD	Pro‐DLB = MCI‐AD	Ciafone et al. (2022)
			18 pro‐DLB	32 MCI‐AD 45 MCI stable	Pro‐DLB = MCI‐AD Pro‐DLB < MCI stable	Yoon et al. (2015)
			67 poss pro‐DLB	56 MCI‐PD	Pro‐DLB = MCI‐PD	Wright et al. (2023)
			148 pro‐DLB	469 no symptom 275 1 symptom	Pro‐DLB = no symptom	Blanc et al. (2022)
			9 pro‐DLB	12 MCI‐AD	Pro‐DLB < MCI‐AD	Jicha et al. (2010)
			21 pro‐DLB	107 MCI‐AD 164 MCI stable	Pro‐DLB < MCI	Sadiq et al. (2017)
			73 pro‐DLB	124 MCI‐AD	Pro‐DLB < MCI‐AD	Van De Beek et al. (2020)
			15 poss pro‐DLB 39 prob. pro‐DLB	31 HC 35 MCI‐AD		Hamilton et al. (2023)
			19 pro‐DLB	25 MCI‐AD	Pro‐DLB = MCI‐AD	Bussè et al. (2018)
			20 pro‐DLB	46 MCI‐PD	Pro‐DLB = MCI‐PD	Yoon et al. (2014)
			49 pro‐DLB	162 MCI‐AD 116 MCI stable	Pro‐DLB = MCI‐AD	Ferman et al. (2013)
			75 DLB (PRO‐DLB + DLB)	91 HC 121 AD + VaD 140 MCI	DLB < controls	Garcia Basalo et al. (2017)
			15 pro‐DLB	13 HC 40 DLB	Pro‐DLB < HC	Jeong et al. (2024)
			143 pro‐DLB	429 MCI‐AD	Pro‐DLB = MCI‐AD	Wyman‐Chick et al. (2025)
			38 pro‐DLB	59 HC	Pro‐DLB < HC	Železníková et al. (2024)
			35 MCI‐DLB	28 HC	Pro‐DLB < HC	Novakova et al. (2024)

Abbreviations: AD, Alzheimer Disease; DLB, Dementia with Lewy Bodies; FTD, Frontotemporal Dementia; HC, Healthy Controls; MCI, Mild Cognitive Impairment; PD, Parkinson Disease; Poss, Possible; Prob, Probable; Pro‐DLB, Prodromal DLB; VaD, Vascular Dementia; VLOSLP + LB, Very Late Onset Schizophrenia Like Psychosis with Lewy Bodies; VLOSLP‐LB, Very Late Onset Schizophrenia Like Psychosis without Lewy Bodies.

The first remark concerns the evaluated cognitive domains from the analysed studies: in addition to global cognitive functioning, attention, executive functions, memory, working memory, visual perception, and language are commonly assessed. An exception is time perception that was examined only in one paper [15]. From the reviewed studies, there were 44 tests that were frequently used to evaluate early presentations of neurodegenerative diseases, 9 of which are organised in multiple subtests, increasing the number to 66 if these are considered individually. Subtests refer to the distinct components into which certain tasks are divided. For example, verbal memory tests typically include immediate recall, delayed recall, and recognition components; visual memory tasks often comprise a copy trial followed by immediate and delayed recall; the Visual Object and Space Perception (VOSP) battery is structured into two sections assessing visual perception and visuospatial abilities, each including several tasks. The most frequently used test is the letter verbal fluency test with 22 studies including this test in their assessment [8, 11, 16, 17, 18, 19, 20, 21, 22, 23, 24, 25, 26, 27, 28, 29, 30, 31, 32, 33, 34, 35], followed by the Trail Making Test (TMT) A and B, used in 19 [17, 18, 19, 20, 24, 25, 26, 29, 31, 33, 36, 37, 38, 39, 40, 41, 42, 43, 44] and 17 [17, 19, 20, 21, 24, 25, 26, 30, 33, 36, 37, 38, 39, 40, 41, 43, 44] studies, respectively. Other commonly used tests were the category verbal fluency test [11, 22, 23, 24, 25, 26, 27, 29, 30, 32, 33, 34, 35, 38, 41, 43, 44] and the digit span backward test [8, 11, 16, 18, 21, 22, 27, 28, 29, 31, 32, 33, 43, 45] used in 17 and 14 studies, respectively. Frequency of use, however, does not imply diagnostic accuracy as further illustrated in the population comparison section.

### 3.3 Impaired Domains

Data inspection allowed us to outline a specific and characteristic neuropsychological profile typical of prodromal DLB patients. Broadly, while performance in memory and language tasks remained almost unaffected during the early stages of the disease, patients showed a significant impairment in performance on tests that evaluate executive functions, attention, and attentional‐executive abilities, that is, those arising from the interaction between the two domains, such as interference suppression or cognitive flexibility. Additionally, working memory and visual perceptual abilities are mildly compromised. For visual perceptual abilities, the extent of impairment varies across subdomains: the visual constructive domain is highly compromised, whereas visuospatial and pure visual perceptual abilities are less affected.

### 3.4 Populations Comparison

**FIGURE 2:**
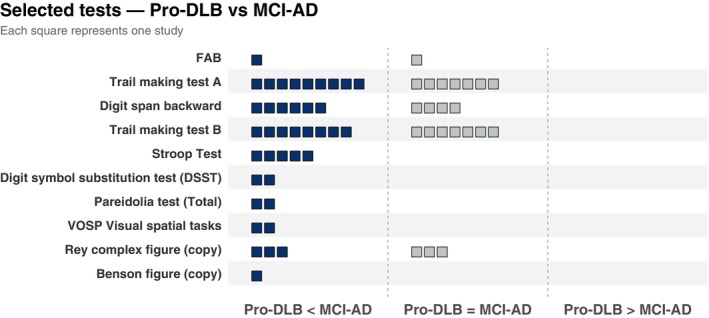
Graphical summary of the most relevant tests sensitive to distinguishing prodromal DLB from MCI‐AD. Each square represents one study. Grey squares indicate comparable performance between groups; blue squares indicate worse performance in prodromal DLB.

Most studies compared a prodromal DLB population with an MCI‐AD population, a control group of HC, or both [8, 15, 16, 17, 18, 19, 20, 21, 22, 25, 26, 27, 28, 29, 30, 31, 32, 33, 34, 35, 36, 37, 38, 39, 40, 42, 43, 46, 47, 48, 49]. Other studies compared prodromal DLB patients with those in various stages of other dementias (vascular dementia, frontotemporal dementia, and others) [24, 41, 44, 45] and with patients with MCI‐PD [11, 23]. Nevertheless, the review primarily focused on the comparison between prodromal DLB and MCI‐AD, as well as the differences between prodromal DLB patients and HC, given that HC comparisons allow the identification of cognitive changes that deviate from normal ageing, while MCI‐AD is the most extensively studied condition and provides a strong basis for differential diagnosis. Notably, the frequency of test administration is, however, not necessarily directly proportional to the accuracy of a test. For instance, the letter verbal fluency test and the category verbal fluency test, although showing slight impairments in prodromal DLB patients compared with HC in some cases, fail to distinguish prodromal DLB from MCI‐AD due to similar performance [50]. There were impairments in some cognitive domains that appear to characterise the neuropsychological profile of prodromal DLB patients and the studies that used tests assessing these areas of cognition generated interesting results. The Trail Making Test A (an attentional test) and the Trail Making Test B (an attentional‐executive test) have precise diagnostic accuracy in differentiating early dementia patients from HC and may provide some discriminatory value between prodromal DLB and MCI‐AD, as findings consistently show either comparable performance or worse performance in prodromal DLB, with no evidence of poorer performance in MCI‐AD. Attentional deficits are potentially related to early involvement of brainstem arousal systems such as the reticular formation [50]. For working memory, the digit span backward test appears to show good discriminatory power among prodromal DLB patients, HC, and MCI‐AD patients [16, 18, 27, 31, 32, 33, 43], highlighting that impairments in this domain are already present in the prodromal stages, possibly reflecting early fronto‐striatal and dopaminergic pathway involvement [50, 51]. Additionally, evaluation of executive functions using the Frontal Assessment Battery (FAB) [11, 22, 27, 37, 46], the Digit Symbol Substitution Test (DSST) [21, 33, 34, 35, 42, 45], and the Stroop test [8, 11, 21, 22, 27, 30, 32] appeared accurate and useful for detecting prodromal DLB, in line with early mesocortical dopaminergic dysfunction affecting frontal networks [51, 52]. The DSST and Stroop test are especially helpful in differentiating frontal cortex impairments more precisely, ensuring the differentiation of prodromal DLB from other dementias. The AX‐CPT, a computerised test that evaluates sustained attention, showed good accuracy specifically in terms of omission errors, although it was used only in one study [28]. Lastly, several tests that assess visuo‐perceptual abilities demonstrated good discriminatory power. Significant results were found for the Rey‐Osterrieth Complex Figure copy [8, 11, 22, 30, 31, 32], outlining visuo‐constructive skills, while the Pareidolia test [38, 47] and the Visual Object and Space Perception Battery (VOSP) [24, 27, 31] also showed high reliability in differentiating between diagnostic groups, consistent with early occipital and parietal–occipital involvement [48, 53]. The copy of the Rey‐Osterrieth Complex Figure is a test that combines praxic‐constructive abilities with visuo‐perceptual ones; it is currently unknown which specific cognitive domain impairment leads to worse performance on this test in the target population. Figure 2 summarises the findings.

**FIGURE 3:**
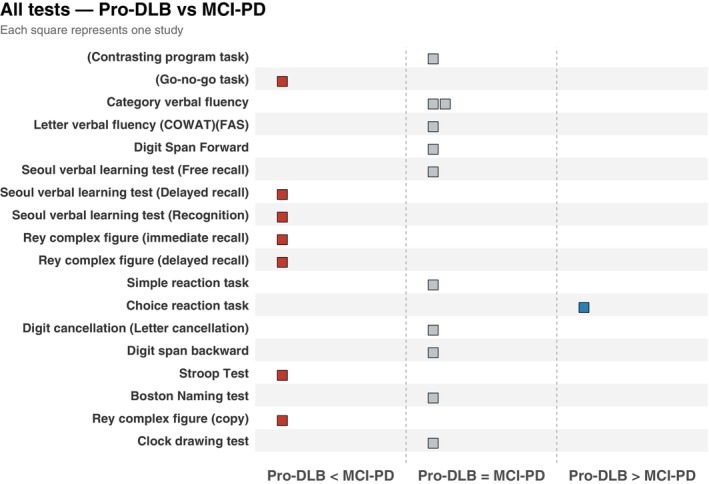
Graphical summary of the comparison between prodromal DLB and MCI‐PD patients across cognitive domains. Each square represents one study; red squares indicate worse performance in prodromal DLB patients; grey squares indicate comparable performance between groups; blue squares indicate better performance in prodromal DLB patients.

Some of the articles included in this systematic review reported comparisons between prodromal DLB groups and patients with MCI due to other neurodegenerative conditions [11, 23, 44]. Patients with MCI‐PD demonstrate an overall cognitive profile broadly comparable with that of prodromal DLB patients across multiple domains. However, notable differences emerge in memory functioning: prodromal DLB patients exhibit significantly poorer performance in long‐term memory tasks [11, 23], indicating a more pronounced impairment in this domain relative to the MCI‐PD cohort. Figure 3 summarises the findings.

**FIGURE 4:**
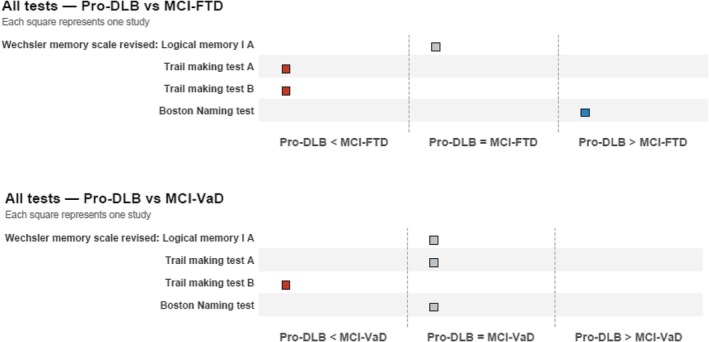
Graphical summary of the comparison between prodromal DLB and MCI‐FTD or MCI‐VaD patients across cognitive tests; each square represents one study; red squares indicate worse performance in prodromal DLB; grey squares indicate comparable performance; blue squares indicate better performance in prodromal DLB patients.

Comparisons involving MCI due to frontotemporal dementia (MCI‐FTD) and MCI due to vascular dementia (MCI‐VaD) are limited, with only one selected study having examined these populations against a prodromal DLB sample [44]. In the prodromal DLB versus MCI‐FTD comparison, prodromal DLB patients performed significantly worse on the Trail Making Test A and B, reflecting deficits in the attention–executive domain, while demonstrating superior scores on the Boston Naming Test, a measure of language production. In the prodromal DLB versus MCI‐VaD comparison, the only significant difference observed was poorer performance of prodromal DLB patients on the Trail Making Test A. Figure 4 summarises the findings.

### 3.5 Quality Assessment

**FIGURE 5:**
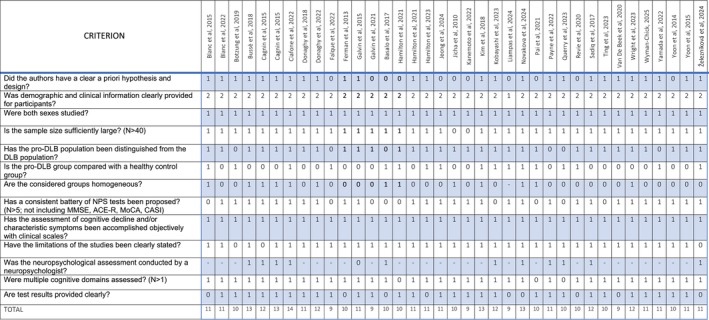
Quality assessment scale.

The methodological quality of all papers was assessed using 13 predefined criteria, using a modified version of the ‘QualSyst’ assessment tool [54]. This checklist was further modified in the present study to reflect better the specific aims and context of the review. We evaluated each study based on population characteristics and how neuropsychological evaluations were carried out by the authors, with a maximum possible score of 14 points (see Figure 5 for quality assessment details), as criterion number 2 could be worth up to 2 points. Overall, all studies have moderate quality, although some lack important information. This can be explained in several ways. Firstly, research in this field is still in its infancy, and authors cannot always rely on significant previous knowledge. Secondly, many studies investigated differences between diseases without including a healthy control group. Lastly, the identification and diagnosis of prodromal DLB are still challenging; often evidence is obtained from studies with discrepant sample sizes, and consequently the resulting analyses are less reliable and not robust.

## 4 Discussion

This systematic review has identified a set of specific tests that appear to be most suitable to detect the earliest cognitive manifestations of Lewy body pathology. The findings of this systematic review suggest that, in prodromal DLB, executive functions, attention, and attentional‐executive abilities are the cognitive domains more severely affected in this population compared with both HC and MCI‐AD. Visual perceptual and visuo‐constructional abilities were also found to be impaired in prodromal DLB. These results are in line with those of previous studies in established DLB [3, 12, 53].

Lewy bodies and α‐synuclein build‐up in diseases with Lewy bodies seems to follow a pattern [51, 52] from which the sequence of characteristic symptoms derives. During the initial phase of accumulation, the brainstem is affected, particularly areas responsible for maintaining vigilance and arousal, such as the reticular formation and its gigantocellular nuclei [50]. Damage to this formation could potentially lead to the attentional fluctuations typical of early DLB [3, 5], as well as the general attentional deficits outlined in this review. Subsequently, Lewy bodies also accumulate in the midbrain, affecting the ventral tegmental area, the origin of the mesocortical dopaminergic pathway, leading to possible frontal dysfunctions [51, 52], reflected in the executive deficits highlighted in this review. Then, the substantia nigra is also damaged [51], possibly leading to degradation of working memory and attentional‐executive abilities [50]; deterioration of these fronto‐striatal pathways could trigger impairments in these domains. Two of the early affected brain areas are the occipital and parietal‐occipital regions, damage of which could be responsible for perceptual deficits in the visual domain, symptoms that were prominently detected in the studies included in the present review. Furthermore, the ventral visual pathway is likely involved in the degeneration, and this could explain the early and frequent occurrence of pareidolias in prodromal DLB patients. Pareidolias, which may be also observed in healthy individuals although more rarely, refer to the perception of meaningful or recognisable images—such as faces—in ambiguous stimuli where none are present. This overinterpretation of neutral images with superimposed symbolic meaning may be a prognostic risk factor for the development of complex visual hallucinations, a typical symptom of patients with established DLB. Altogether, the diverse symptomatology observed in prodromal DLB closely reflects the Lewy body‐related neurodegeneration identified through neuroimaging [52], establishing a strong link between the neuropsychological profile and the underlying neuropathology.

In this review we also identified the tests that best differentiated prodromal DLB from HC and MCI‐AD. In brief, for executive functions the digit span backward test, the Frontal Assessment Battery (FAB), the Digit Symbol Substitution Test (DSST), and the Stroop test were found to discriminate prodromal DLB patients from HC and MCI‐AD patients. For attention, the TMT‐A and TMT‐B appeared to distinguish prodromal DLB from HC and partially from MCI‐AD. Finally, for visuo‐perceptual domains the Rey‐Osterrieth Complex Figure copy, the Pareidolia test and the Visual Object and Space Perception Battery (VOSP) showed good differentiation. Among the VOSP tasks, those assessing spatial tasks (Dot Counting, Position Discrimination, Number Location, and Cube Analysis) yielded better results than those evaluating visual perception (fragmented letters, silhouettes, object decision, progressive silhouettes).

Some of the studies included in this review reported comparisons with patients experiencing MCI due to other forms of neurodegeneration [11, 23, 44]. Comparisons with MCI‐PD, for example, highlighted a broadly similar cognitive profile across multiple domains, with the notable exception of long‐term memory, where prodromal DLB patients showed significantly greater impairment [11, 23]. Comparisons with MCI‐FTD revealed relatively worse performance in prodromal DLB on attentional‐executive measures (TMT A and B), alongside better performance on a language production task (Boston Naming Test) [44]. In contrast, the comparison with MCI‐VaD yielded only a single significant difference, again in attentional processing (TMT A), with prodromal DLB patients performing more poorly [44]. The findings are, however, based on a small number of cases and with heterogeneous assessments and, therefore, can only provide exploratory indications that need confirmation in larger empirical studies [11, 23, 44]. These results are broadly in line with those of a recent meta‐analysis [12] that aimed at identifying the cognitive domains and neuropsychological tests that best distinguish MCI‐LB from HC and MCI‐AD. This meta‐analysis highlighted a prominent impairment of attention/processing speed and attention/executive functions in MCI‐LB, with a more modest role for visuospatial dysfunction. The largest effect size was observed for TMT A and B when comparing MCI‐LB and MCI‐AD indicating that scores on both the Trail Making Tests A (testing attention/processing speed) and B (testing attention/executive abilities with a processing speed component) sections perform well in distinguishing MCI‐LB from MCI‐AD. These findings are only partially consistent with the current systematic review. Our results, indeed, indicate a more variable pattern, with studies reporting either comparable performance or poorer performance in prodromal DLB, suggesting a less robust and less consistent discriminatory value. The reasons underlying the differences in these results likely lie in several methodological differences: our review included a larger number of studies that had used the TMT A and B, and there was greater heterogeneity in the diagnostic criteria used across these studies compared with the meta‐analysis. Specifically, we analysed 19 studies using the TMT A and 17 using the TMT B, of which only 7 (TMT A) and 6 (TMT B) overlap with those included in the meta‐analysis. In addition, the selection of inclusion and exclusion criteria differed. Notably, unlike the meta‐analysis, we excluded studies in which the target population consisted of iRBD patients. This difference in study populations may have contributed to the discrepancies in results. Additionally, unlike the meta‐analysis, this review includes a formal quality assessment of the studies, enhancing the interpretability and reliability of the findings. Beyond these methodological differences, the present review not only provides a broader overview of neuropsychological tests currently used in clinical settings but also aims to propose innovation within a field that is continually evolving. It is among the first to integrate the neuropsychological profile of prodromal DLB with its underlying neuropathological progression, aligning the most consistently impaired cognitive domains with the corresponding neurophysiological changes described in the literature. This approach highlights the temporal relationship between histopathological alterations and emerging cognitive deficits, and supports the proposal of a new targeted, structured neuropsychological battery. In addition, the current review included studies that report comparisons between prodromal DLB patients and patients with MCI due to other forms of non AD neurodegeneration, enhancing, therefore, the specificity of its findings.

Indeed, based on the findings of the present review, a possible neuropsychological battery more specific and sensitive to the identification of individuals in the prodromal stage of DLB can be suggested. This battery should include tests that evaluate attentional and executive abilities, working memory capacity, as well as tests assessing the entire visual perceptual domain, particularly visuo‐constructive and visuo‐perceptual aspects. Among the currently available tests, selected subtests of the VOSP (i.e., cube analysis), Stroop test, digit span backward, Rey‐Osterrieth Complex Figure copy (or the Taylor figure copy), DSST, pareidolia test, and FAB could be included in this battery. However, further research is required to establish the early impairment of these cognitive functions and to support the validation and standardisation of neuropsychological tests specifically designed for the assessment of these patients and the early detection of the disease.

### 4.1 Limitations

Even though the study was conducted following strict criteria, it is important to acknowledge some limitations. First, one of the inclusion criteria specified that patients with iRBD should not be included, as this symptomatology may not necessarily align with the prodromal stage of dementia with Lewy bodies (pro‐DLB) since a large proportion of patients with RBD might not develop DLB [13, 14]. However, if future research establishes a definitive link between the two conditions, this systematic review will have overlooked an important body of literature and would need to be updated. Another limitation concerns the search strings used to identify relevant articles; some pertinent studies may have been excluded, as using the same terms on other databases, different from PubMed and Web of Science, could yield different results. A third limitation is that some researchers used standardised neuropsychological test batteries, without differentiating them based on the diagnostic categories of the target population. As a result, some of the tests included in this review are commonly used but not necessarily intended to identify pro‐DLB patients or distinguish them from other populations. An additional limitation pertains to the clinical criteria for diagnosing pro‐DLB; while the McKeith et al. criteria [3] are now widely recognised as the gold standard, some of the included studies used older criteria that were acceptable at the time but are no longer in use. This variation across studies emphasises the diagnostic inconsistency in the identification of pro‐DLB that may have led to diagnostic errors in earlier research. Finally, article screening for title only was carried out by a single reviewer. To mitigate the risk of rejecting suitable articles, rejection was done on title only when this was unequivocal about the content of the article. If the content of the article could not be clearly determined based on title only, this automatically led to additional screening of the article abstract. This conservative strategy minimised the potential for inaccurate rejections at this level of the screening process.

## 5 Conclusions

The diagnosis of prodromal dementia with Lewy bodies remains challenging, but there has been significant progress in more recent years. One key advancement for the future is the development of tools that can differentiate various dementia phenotypes in the early stages, allowing the identification of pro‐DLB patients. This review makes a significant contribution to the difficult process of creating a comprehensive battery of neuropsychological tests specifically designed for the diagnosis of prodromal DLB. It has drawn attention to tests used to evaluate specific cognitive domains, emphasising the potential to create a battery that focuses solely on these functions, while highlighting the need to update tests that are currently outdated. The goal of this effort is not only to differentiate between various dementia aetiologies but also to enhance, optimise, and anticipate the clinical and pharmacological care provided to these patients.

## Author Contributions

**Francesco Mori:** conceptualisation, methodology, writing – original draft, data curation, formal analysis. **Fabrizia D'Antonio:** conceptualisation, writing – review and editing, methodology. **Giulia Zazzaro:** writing – review and editing, data curation. **Desirée Conti:** writing – review and editing, data curation. **Micaela Mitolo:** writing – review and editing. **Annachiara Cagnin:** writing – review and editing. **Annalena Venneri:** conceptualisation, methodology, supervision, writing – review and editing.

## Funding

A.V. is supported by funding obtained under the National Recovery and Resilience Plan (NRRP), Mission 4 Component 2 Investment 1.3—Call for tender No. 341 of 15/03/2022 of the Italian Ministry of University and Research funded by the European Union‐NextGenerationEU, Project code PE0000006, Concession Decree No. 1553 of 11/10/2022 adopted by the Italian Ministry of University and Research, CUP D93C22000930002, ‘A multiscale inte‐grated approach to the study of the nervous system in health and disease’ (MNESYS).

## Ethics Statement

The authors have nothing to report.

## Conflicts of Interest

The authors declare no conflicts of interest.

## Data Availability

There are no new data generated for this manuscript.
